# Neurology

**Published:** 1905-04-22

**Authors:** 


### NEUROLOGY.

*(Continued from page 54.)*

**Traumatic Hæmorrhage over Broca's Convolution.**—A most remarkable case of intra-cranial hæmorrhage due to contrecoup has been published by Krauss.[4] A man, aged 62, fell out of a tramcar on August 10th, striking the right side of his head and right shoulder. He was a little stunned, but said he was "all right" and was driven home. The right eyelid was then found swollen and discoloured. On August 11th he was, but for the bruises, normal. On August 12th he began to jabber in an unintelligent and incoherent manner; the first two or three words of a sentence being distinctly spoken whilst the remainder was an unintelligible jumble. During the day he became more confused, but examination showed no distinct evidence of any cerebral lesion. The next day he was more stuporous. On the following day, August 15th, the patient began to have convulsive seizures of the right side of the face, with jerking of the tongue to the right. There was no involvement of the right arm or leg. The patient was trephined over the third left frontal convolution; no blood was found between the bone and dura, but between the latter and the pia blood clots were found—to the amount of about three teaspoonfuls—and evacuated. Primary union took place. Two convulsive seizures took place the first night, but there was none subsequently, and the patient got quite well. What happened was a small localised hæmorrhage of the brain membranes on the side of the head opposite to the blow—a contrecoup hæmorrhage. This occurred immediately over Broca's convolution, but did not produce any difficulty of speech until some 60 hours after the injury; its slow increase brought about aphasia and paraphasia, and later on facial epilepsy of the Jacksonian type.

**Juvenile Tabes.**—A most interesting case of juvenile tabes has been published, with comments, by Grinker.[5] The family history was complete. The father suffered from syphilis, and died at the age of 47 from some pulmonary complaint. The mother contracted syphilis from her husband, and subsequently developed tabes, which was verified by post-mortem examination. Of their three children, a son died from general paralysis at the age of 19; a daughter, still living, has suffered from snuffles, chronic hydrocephalus, condylomata, disseminated choroiditis, interstitial keratitis, and hemiplegia probably due to thrombosis; and a son, now aged 25, suffers from juvenile tabes. As an infant he had condylomata and skin rashes. The first symptoms of tabes were optic atrophy in the right eye, occurring at the age of 16, followed by that of the left a year later, and a little subsequently by lightning pains. At the present time he has loss of tendon jerks, ataxia, rigid and unequal pupils, optic atrophy, ptosis, lightning pains, bladder disturbances, girdle sensation, and paræsthesia. This case cannot be one of Friedreich's ataxia; for in that disease the ataxia is cerebellar in type, and occurs both at rest and in motion, there is a chorea-like oscillation of head and trunk, the arms are early involved, the knee-jerks are lost late, there are no lightning pains, no optic atrophy or Argyll-Robertson pupil, there are scanning speech, nystagmus, scoliosis, club-foot, and disturbances of intellect. Such cases of true juvenile tabes are rare, for most reported have been in reality cases of Friedreich's ataxy. It agrees with the others recorded in that the early symptoms were optic atrophy, vesical trouble, and sensory disturbances, and in the presence of syphilis in the parents. Opinions are varied as to which is the more important, the soil or the morbific agent, in producing nervous disease subsequent to syphilis. Some French authorities suggest that there is a special form of syphilis affecting preferably the nervous system, basing that view on cases of conjugal tabes, and on cases of nervous syphilis derived from a single source and resulting in the production of identical symptoms. In the particular case reported here it is not possible to say which was the more important etiologic factor, the neuropathic family tendency, or the specific syphilitic nerve-virus. It might be cited as supporting the views of Marie and others above referred to.

**Tender Spots on the Spine.**—Shadwell[6] has recorded a number of cases in which pain in various parts of the body was complained of, and on examination tender spots at the point of origin of the spinal nerves supplying the region of the body were found, and by treating these complete and permanent relief was the result. For instance, a woman aged 26 complained of severe pain in the left side of her chest, made worse by deep breathing. Poultices, liniments, and blisters were applied to the chest but gave little or no relief. A fortnight later, careful examination showed that there was no pleurisy or lung trouble, but it was found that a tender spot existed over the seventh dorsal vertebra, causing the patient to flinch when it was pressed upon. She had been unconscious of any pain in the spine. This painful spot was painted with linimentum iodi, with the result that the pain in the side of the chest disappeared; there was no return, and the patient remained well. Another case is the following: a woman, aged 46, came complaining of pain extending up the back of the head, so severe that she held her head with both hands and rocked from side to side as she sat in her chair. There was a very tender spot over the upper cervical vertebræ. A blister was applied. On the next day the pain was much better, and on the third day it was gone. The nature of this and similar cases is doubtful; but it is to be noted that in this class of cases the patients never complain of pain in the spine; further, in herpes zoster and intercostal neuralgia the spine is not tender and no relief follows the application of a blister over the spine.

[4] Amer. Jour. Med. Sci., Sept. 1904. [5] Jour. of Nerv. and Ment Dis., Dec. 1904. [6] Lancet, Sept. 10, 1904.

